# MCGCL:Adversarial attack on graph contrastive learning based on momentum gradient candidates

**DOI:** 10.1371/journal.pone.0302327

**Published:** 2024-06-06

**Authors:** Qi Zhang, Zhenkai Qin, Yunjie Zhang

**Affiliations:** 1 School of Computer Science and Technology, Soochow University, Suzhou, Jiangsu, China; 2 Guangxi Police College, Nanning, Guangxi, China; University of California Los Angeles, UNITED STATES

## Abstract

In the context of existing adversarial attack schemes based on unsupervised graph contrastive learning, a common issue arises due to the discreteness of graph structures, leading to reduced reliability of structural gradients and consequently resulting in the problem of attacks getting trapped in local optima. An adversarial attack method based on momentum gradient candidates is proposed in this research. Firstly, the gradients obtained by back-propagation are transformed into momentum gradients, and the gradient update is guided by overlaying the previous gradient information in a certain proportion to accelerate convergence speed and improve the accuracy of gradient update. Secondly, the exploratory process of candidate and evaluation is carried out by summing the momentum gradients of the two views and ranking them in descending order of saliency. In this process, selecting adversarial samples with stronger perturbation effects effectively improves the success rate of adversarial attacks. Finally, extensive experiments were conducted on three different datasets, and our generated adversarial samples were evaluated against contrastive learning models across two downstream tasks. The results demonstrate that the attack strategy proposed outperforms existing methods, significantly improving convergence speed. In the link prediction task, targeting the Cora dataset with perturbation rates of 0.05 and 0.1, the attack performance outperforms all baseline tasks, including the supervised baseline methods. The attack method is also transferred to other graph representation models, validating the method’s strong transferability.

## Introduction

Graph neural networks have gained widespread application across various domains due to their outstanding feature extraction mechanisms and excellent predictive performance. These domains include social networks [1, 2], recommendation systems [3, 4], and traffic prediction [5, 6]. By aggregating information from neighboring nodes, these networks enable models to effectively learn the underlying graph structure, leading to significant improvements in downstream tasks such as graph classification [7, 8], node classification [9], and link prediction [10, 11].

However, the application security of graph neural networks has raised concerns due to their weak interpretability and the discrete complexity of graph data. Recent research indicates that graph neural networks are susceptible to adversarial attacks [12], where attackers can deliberately insert imperceptible perturbations into the input, potentially causing prediction errors in the model and increasing the potential risks of graph neural network applications. For instance, attackers could forge transaction data in a banking system, leading to fraud detection models misclassifying fraudulent transactions as normal ones, resulting in unforeseen consequences.

By delving into the study of graph adversarial attacks, we can examine the issue from the attackers’ perspective, uncovering inherent security vulnerabilities in neural networks and, more effectively, preventing potential risks. This research enhances the robustness of graph neural networks, ensuring their security and reliability in practical applications. Adversarial attacks have been extensively studied in the field of deep learning [13, 14].

In graph research, numerous studies have explored the robustness of graph data. The generation of adversarial samples for graph data primarily relies on the saliency of structural gradients, i.e., the gradient information of the objective function concerning the adjacency matrix. Nettack [15] first proposed graph adversarial attacks, targeting specific nodes in the graph by introducing perturbations or noise through modifications to the graph structure (such as adding or removing edges) or node attribute features (such as adding or modifying attributes), resulting in misclassification of the targeted node by the model. Metattack [16] constructed an adversarial attack model using meta-learning methods, simultaneously considering the meta-learning process of graph neural networks. Xu et al. [17] introduced a topology attack method based on an optimization perspective. This method initially optimizes the graph, generating a series of topology attack samples, and subsequently utilizes these samples to attack graph neural networks. The paper also introduces projection gradient descent (PGD) and Min-Max strategies for optimizing topology attacks.

Gradients have been widely applied as a critical basis for generating perturbations. However, researchers have started to investigate the impact of the reliability and saliency of gradients on perturbation updates. Liu et al. [18], by studying the gradient errors caused by model optimization uncertainty and robustness discussed the role of gradients in graph adversarial attacks. They introduced features like momentum gradients and added Gaussian noise to reduce the impact of gradient uncertainty. EpoAtk [19] demonstrates that the discreteness of graph structures leads to the fact that the most significant gradients do not necessarily have the maximum impact on the model predictions. The paper proposes an exploratory method to alleviate local optima issues while generating perturbation graphs.

Currently, adversarial attacks on graph neural networks primarily rely on supervised methods [20, 21]. These methods heavily rely on accurate labels and are tailored for specific downstream tasks, while in reality, there is a substantial amount of unlabeled data. The costly nature of labeled data and graph structure complexity have become bottlenecks in developing supervised adversarial attacks.

Due to the high dependence on labeled samples, semi-supervised and unsupervised graph representation learning has become a widely studied focus [22–24]. Graph contrastive learning, as an unsupervised graph representation learning method, has been widely studied and applied in many practical scenarios due to its excellent feature extraction ability [25].

However, exploring the robustness of unsupervised graph representation learning remains challenging [26]. Adversarial attacks and defenses for unsupervised graph neural networks have become a hot topic in current research. Bojchevski et al. [27] conducted the initial study on unsupervised graph adversarial attacks, leveraging a property in generating embeddings using a random walk-based algorithm in spectral graphs. The study reduced the effectiveness of embeddings for newly generated graphs by modifying a limited number of edges. However, this method only applies to the DeepWalk model [28], and its generality and effectiveness on other graph neural networks and downstream tasks are limited.

Zhang et al. [29] employ ordinary gradients of the contrastive loss concerning the adjacency matrix of the data-augmented graph in adversarial attacks. After summing the gradients of the two views, they directly choose the edge corresponding to the most significant gradient value as the perturbed edge for the model attack, achieving specific adversarial effects. However, due to the discreteness of graph structures and the neglect of the reliability factor of gradients, this method is prone to local optima, resulting in suboptimal perturbation effects and slow convergence speed.

In order to solve the above challenges, an adversarial attack method on graph contrastive Learning based on momentum gradient candidates(MCGCL) is proposed in this paper. Specifically, after obtaining the node gradient matrix, a certain proportion of the previous gradient information is added to transform ordinary gradients into momentum gradients, thereby improving the convergence speed and accuracy of gradient updates. Subsequently, the gradients of two views are summed, and based on the candidate set size, gradient saliency, and perturbation rules, a candidate perturbation graph set is generated. All candidate perturbation graphs are assessed using an evaluation function, and the final perturbed edge is selected by comparing the evaluation results. This process aims to generate adversarial samples with more effective perturbation effects, enhancing the attack’s effectiveness. Experimental results demonstrate the method’s efficacy and transferability.

The main contributions based on this article are summarized as follows.

- Due to the discreteness of the graph data and the uncertainty of model parameter updates, the use of momentum gradients instead of ordinary gradients is proposed. The momentum gradients utilize previous gradient information to guide gradient updates, improving the stability and efficiency of the optimization process. Improve the accuracy of parameter updates and model convergence speed, improving attack effectiveness and reducing resource waste.
- Due to the non-Euclidean graph structure and the noise introduced by data augmentation, the most significant gradient values may have a lower impact on model predictions. This paper proposes an exploratory method based on a contrastive model for candidate selection and evaluation. This method mitigates the risk of adversarial attack results falling into local optima, generating more perturbed and impactful adversarial samples. The method enhances the accuracy of perturbations by reducing the risk of being trapped in local optima, thus improving the overall effectiveness of the generated adversarial samples.
- The proposed method is tested on three real datasets and two downstream tasks and is compared with various supervised and unsupervised attack methods. Extensive experiments demonstrate the effectiveness of our method. Furthermore, the attack method is extended to different graph contrastive learning models and other classical graph neural networks, confirming the strong transferability of our method. The effectiveness of the two proposed strategies is validated through ablation experiments.

## Related works

### Graph adversarial attack

Security concerns have become a focal point of attention with the rapid development and widespread application of neural networks. Designing efficient and broadly applicable adversarial attack and defense [30, 31] strategies has emerged as a current research hotspot. Existing attacks can be classified into several categories based on different criteria.

Based on different attack stages, attacks can be categorized into node poisoning attacks before model training [27] and evasion attacks during model training or testing stages where the attacker cannot modify model parameters and structures [32]. Depending on the attacker’s knowledge of the target model, attacks can be classified into black-box attacks [33], grey-box attacks [34], and white-box attacks [35]. According to different attack objectives, attacks can be further divided into targeted [15] and global attacks [16].

The attack scenario in this paper involves grey-box global poisoning attacks. Attackers leverage training data to train substitute models, allowing them to infer information from the victim model. Since altering node features has a limited impact on the perturbed graph structure and some graphs lack node features, such as the PolBlogs dataset, this paper focuses solely on perturbing the topological structure. The goal is to reduce the overall predictive performance of the model, aligning with the practical requirements of real-world applications.

### Graph contrastive learning

Graph contrastive learning is a typical representation of unsupervised graph neural networks. DGI [36] is a pioneering study in graph contrastive learning, introducing an unsupervised learning method that maximizes the mutual information of graph data to learn node representations. GraphCL [37], during the data augmentation process, incorporates a comprehensive set of random augmentation strategies, considering both topological structure and node features. On the other hand, the contrastive learning model with a negative sample sampling strategy [38] effectively converted all nodes except the positive sample into negative samples by selecting nodes with labels different from the center node. In addition, it utilized GCN [39], SGC [40], and APPNP [41] as shared graph neural network models.

This paper adopts GCA [42] as the framework for contrastive learning. On the topological level, an enhancement scheme based on node centrality metrics highlights important connectivity structures. On the node attribute level, more noise is added to the features of less important nodes to disrupt node features, thereby forcing the model to recognize underlying semantic information. This paper’s data augmentation combines topological and feature levels, sharing a graph neural network with two layers and a Multilayer Perceptron(MLP). The graph embeddings generated through graph contrastive learning can be applied to downstream tasks such as node classification (predicting node categories) and link prediction tasks (predicting potential edges between nodes). The two-layer graph convolutional network(GCN) [39] structure is as follows:

$$\begin{array}{c} Z=f\left(X,A\right)=softmax(\hat{A}ReLU\left(\hat{A}XW^{\left(0\right)}\right)W^{\left(1\right)}), \end{array}$$ (1)

$$\begin{array}{c} Where,\;\hat{A}={\tilde{D}}^{-\frac{1}{2}}\tilde{A}{\tilde{D}}^{-\frac{1}{2}}. \end{array}$$

Among them, $\tilde{A}$ represents the adjacency matrix with self-loops, where $\tilde{A}=A+I$. ${\tilde{D}}_{ii}=\sum_{j}{\tilde{A}}_{ij}$ denotes the degree matrix. The activation functions used are *softmax* and *ReLU*. The model parameters are denoted as *W*^(0)^ and *W*^(1)^.

$$\begin{array}{c} l\left(u_{i},v_{i}\right)=-log\frac{e^{\beta (u_{i},v_{i})/\tau }}{e^{\beta (u_{i},v_{i})/\tau }+\sum_{k\neq i}\left(e^{\beta (u_{i},v_{k})/\tau }+e^{\beta (u_{i},u_{k})/\tau }\right)}, \end{array}$$ (2)

$$\begin{array}{c} \Gamma =\sum_{i=1}^{N}\left[l\left(u_{i},v_{i}\right)+l\left(v_{i},u_{i}\right)\right]. \end{array}$$ (3)

The contrastive loss function is defined as shown in Eq (2). Here, the similarity measure utilizes cosine similarity, where *β* represents the cosine similarity function and *τ* is the temperature coefficient. The numerator computes the similarity of positive pairs of node embedding representations, while the denominator summarizes the similarity of positive and negative pairs. To reflect the symmetry and balance of the loss, this paper sum both *l*(*u*_*i*_, *v*_*i*_) and *l*(*v*_*i*_, *u*_*i*_).

## Preliminaries

### Notations

In the graph G(V, E), *V* represents the set of nodes with a total of n nodes, and *E* represents the set of edges. *A* ∈ *R*^*N*×*N*^ denotes the adjacency matrix used to represent the graph’s topological structure, where 0 indicates no edge between nodes, and 1 indicates the presence of an edge between nodes. *X* ∈ *R*^*N*×*s*^ represents the feature vectors of the nodes in the graph, where each node corresponds to an s-dimensional feature vector. The symbol definitions used are provided in Table 1.

**Table 1:** Symbols and definitions.

Symbol	Definition
*G*	Original graph
$\hat{G}$	Perturbed graph
*T*	Random augmentation function set
*V*	Node set
*E*	Edge set
*A*	Adjacency matrix of original graph
*A*_1_,*A*_2_	Adjacency matrix of graph augmentation views
*X*	Node feature matrix of original graph
*X*_1_,*X*_2_	Node feature matrix of graph augmentation views
*A*′	Perturbed adjacency matrix
*f*_*θ*_	Graph neural network with parameter *θ*
*θ*′	Model parameters after perturbation
L	Loss function
*σ*	Perturbation budget
*S*	Candidate set size
*p*	Momentum coefficient
Δ_*mt*1_, Δ_*mt*2_	Momentum gradients of graph augmentation views

### Threat model

#### Attacker’s goal

Before model training, perturbation is applied to the dataset by introducing noise, aiming to diminish the overall performance of the learned unsupervised graph contrastive learning model, consequently leading to misclassification in test results.

#### Attacker’s capability

This paper investigates grey-box global poisoning attacks in graph adversarial scenarios. In real-world scenarios, attackers typically lack direct access to the target model. Instead, they leverage training data to train substitute models, enabling them to infer information from the compromised model. Additionally, this study adopts an unsupervised learning method, eliminating the need for access to data labels during the training process.

### Problem definition

Graph adversarial attacks should be regarded as a bi-level optimization problem [43], with the inner loop involving training the graph contrastive learning algorithm and the outer loop focusing on generating perturbed graphs. The following equation defines the problem formulation for node-level graph adversarial attacks.

$$\begin{array}{c} \begin{array}{c} \text{max}\;L_{atk}\left(f_{\theta }\left(\hat{G}\right)\right)=\sum_{u\in V_{t}}l_{atk}\left(f_{\theta^{\prime }}{\left(\hat{G}\right)}_{u},y_{u}\right),\\ s.t.\;\theta^{\prime }=\text{arg}\;\underset{\theta }{\text{min}}\;L_{train}\left(f_{\theta }\left(G^{\prime }\right)\right). \end{array} \end{array}$$ (4)

*G*′ can be *G* or $\hat{G}$. The attacker’s objective is to find a perturbed graph $\hat{G}$ that maximizes the loss value of the nodes, thereby reducing the overall prediction performance of the model.

The attack consists of two main steps: generating adversarial samples and attacking the model. The graph structure matrix is treated as a hyperparameter in generating adversarial samples. Forward propagation is employed to compute the embedding representations of graph nodes and the target loss. Backpropagation is then utilized to calculate the gradient information for all graph nodes under the current model. This gradient vector reflects the contribution of each node to the model’s prediction results. The gradient formula is represented as follows:

$$\begin{array}{c} A_{i,j}^{grad}=\frac{\partial L_{train}}{\partial A_{i,j}}{|}_{A}. \end{array}$$ (5)

Arrange the obtained node gradients in descending order. The attacker determines the perturbation status of edges based on the saliency of node gradients and the actual existence of edges.The specific perturbation rules are as follows:

$$\begin{array}{c} \left\{ \begin{array}{cccc} & (V,E\cup (u,v)), & & A_{i,j}=0\cap A_{i,j}^{grad}>0,\\ & (V,E\backslash (u,v)), & & A_{i,j}=1\cap A_{i,j}^{grad}<0. \end{array}\right. \end{array}$$ (6)

*E* ∪ (*u*, *v*),*E*\(*u*, *v*) represent adding and removing edges, respectively. Under the premise of perturbation rules, select the edge corresponding to the most significant gradient value as the perturbation edge for this iteration. Flipping edge *E*_*i*,*j*_ is a potential perturbation that negatively impacts the victim model. This perturbation is added to the perturbation vector determined in the previous iteration to generate the perturbation graph for this iteration. The attacker optimizes the perturbation vector to minimize the model’s predictive accuracy. The generation of perturbation graphs employs a greedy strategy, perturbing one edge at a time. The schematic diagram is as follows:

$$\begin{array}{c} G^{\left(0\right)}\to G^{\left(1\right)}\to \cdots \to G^{\left(k\right)}\to G^{\left(k+1\right)}\to \cdots \to G^{\left(K\right)}. \end{array}$$ (7)

It is necessary to set a perturbation threshold to ensure the imperceptibility of adversarial samples while meeting the requirement of reducing the overall model performance. The perturbation includes both topological structure perturbation and node attribute feature perturbation. The formula is defined as follows:

$$\begin{array}{c} ||A-A^{\prime }{\left|\right|}_{0}+||X-X^{\prime }{\left|\right|}_{0}\leq 2\sigma . \end{array}$$ (8)

## Method

### Overall

**Fig 1:**
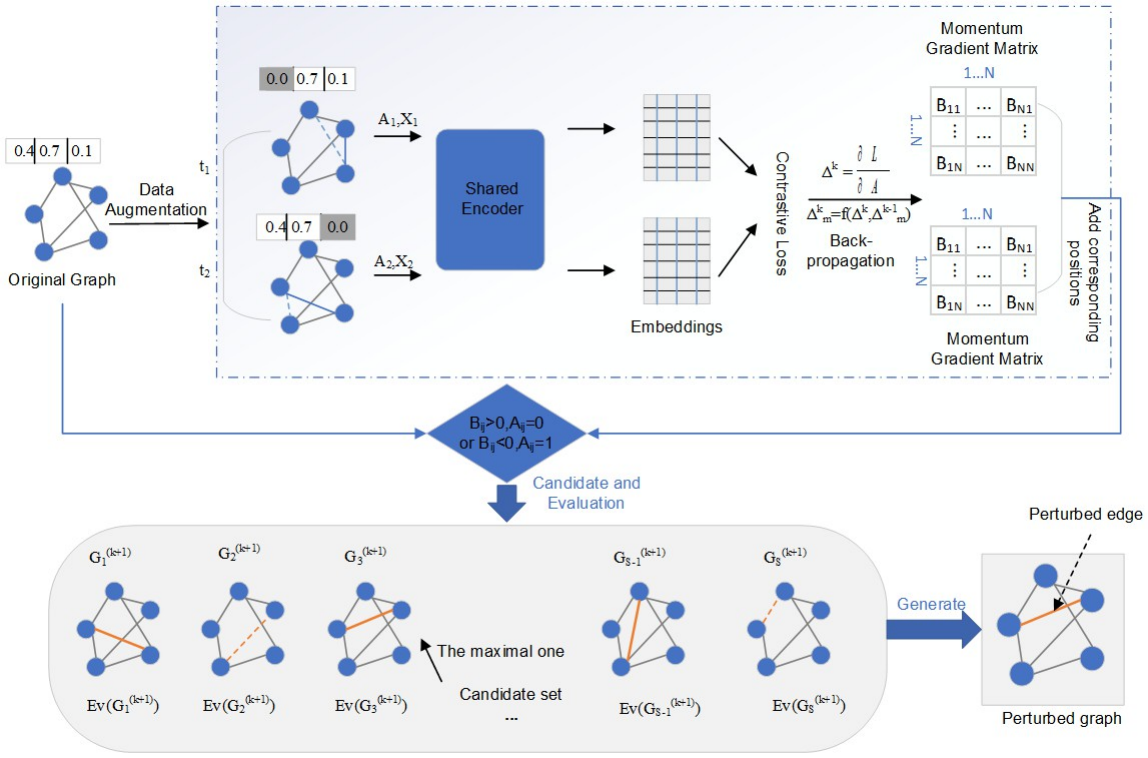
Illustration of MCGCL.

Fig 1 illustrates the framework structure for implementing contrastive learning adversarial attacks using momentum gradient candidates. The original graph undergoes adaptive data augmentation to generate two views. These generated views are input into a shared graph neural network to obtain node embedding representations for each view and compute the contrastive loss. Subsequently, during the backpropagation process, gradients of the loss concerning the adjacency matrices of the two views are calculated separately. Building upon the current gradients, momentum gradients are generated by incorporating a certain proportion of the previous gradients. Subsequently, the absolute values of the momentum gradients are calculated and arranged in descending order, entering the candidate phase. Candidate edges are selected based on the gradient saliency, the candidate set size and the perturbation rule defined in Eq (6), forming a candidate perturbation graph set. After evaluating each candidate graph with an evaluation function, the edge with the highest loss value is chosen as the perturbed edge for this iteration. This process is repeated until the number of perturbed edges reaches the threshold. The objective function is defined as:

$$\begin{array}{cc} & {\text{max}}_{A^{\prime }}\;L\left(f_{\theta^{\prime }}\left(A_{1},X_{1}\right),f_{\theta^{\prime }}\left(A_{2},X_{2}\right)\right), \end{array}$$ (9)

$$\begin{array}{c} \begin{array}{c} s.t.\;\theta^{\prime }=\underset{\theta }{\text{arg}\;\text{min}}\;L\left(f_{\theta }\left(A_{1},X_{1}\right),f_{\theta }\left(A_{2},X_{2}\right)\right),\\ \left(A_{1},X_{1}\right)=t_{1}\left(A^{\prime },X\right),\left(A_{2},X_{2}\right)=t_{2}\left(A^{\prime },X\right),||A-A^{\prime }{\left|\right|}_{0}=2\sigma . \end{array} \end{array}$$

L represents the contrastive loss, *θ* and *θ*′ are the model parameters before and after perturbation, *t*_1_ and *t*_2_ are two randomly chosen augmentation functions. The constraint aims to balance the imperceptibility and effectiveness of the perturbation. The term 2*σ* indicates the same edge for symmetric positions about the main diagonal in the adjacency matrix. Additionally, this paper only perturbs the topological structure while keeping the node attributes unchanged to perturb the entire graph structure. Each view’s gradient is represented as:

$$\begin{array}{c} \Delta_{1}^{k}=\frac{L}{A_{1}^{k}},\Delta_{2}^{k}=\frac{L}{A_{2}^{k}}. \end{array}$$ (10)

$\Delta_{1}^{k}$ and $\Delta_{2}^{k}$ respectively represent the gradients of the loss concerning two views in the k-th iteration.

### Momentum gradients

The gradient is a critical factor in guiding the generation of perturbation vectors. The discreteness of graph data and the uncertainty of model parameter update will cause unreliable factors in the structural gradient. Meanwhile, the perturbation process of edges is iterative, and the perturbed edges generated in each iteration will affect the topological structure of the entire graph. The calculation of momentum gradient uses a weighted sum method, which guides the update of gradients by utilizing previous gradient information, reducing the oscillations phenomenon of gradients during the update process. Therefore, using momentum gradients instead of ordinary gradients can improve the speed of optimization convergence and the reliability of structural gradients, thereby increasing the success rate of attacks.

Specifically, this article proposes to guide gradient updates by accumulating previous gradient information in a particular proportion based on the two view gradient vectors generated by backpropagation. This accumulated gradient is called the momentum gradient, and the specified proportion is called the momentum coefficient, denoted as *p*. The formula for gradient update is as follows:

$$\begin{array}{c} \Delta_{m1}^{k}=\Delta_{1}^{k}+p*\Delta_{m1}^{\left(k-1\right)},\Delta_{m2}^{k}=\Delta_{2}^{k}+p*\Delta_{m2}^{\left(k-1\right)}. \end{array}$$ (11)

$\Delta_{m1}^{k}$ , $\Delta_{m2}^{k}$ are two views of the *k*-th iteration of momentum gradients respectively, $\Delta_{m1}^{\left(k-1\right)}$, $\Delta_{m2}^{\left(k-1\right)}$ are two views of the (*k* − 1)-th iteration of momentum gradients respectively, $\Delta_{1}^{k}$,$\Delta_{2}^{k}$ two views of the *k*-th iteration of gradients respectively.

After data augmentation, differences arise between views and the original graph, impacting the saliency of gradients due to stochastic augmentation methods. The saliency of individual view momentum gradients cannot fully represent the saliency of the original graph gradients. To adequately capture the saliency of the original graph gradients and mitigate biases introduced by stochastic augmentation, this paper utilizes the combined saliency of gradients from two views to determine perturbed edges. The specific formula is expressed as follows:

$$\begin{array}{c} \Delta_{sum}=\Delta_{m1}^{k}+\Delta_{m2}^{k}. \end{array}$$ (12)

Subsequently, the absolute values of the momentum gradients are calculated and arranged in descending order, entering the candidate phase.

### Candidate and evaluation

Gradient saliency characterizes the importance of edges in model prediction. However, due to the discreteness of the graph structure and the presence of data augmentation noise, the most significant gradient may have a small impact on model prediction, leading to the risk of attack results falling into local optima. Therefore, this article proposes an exploratory method for candidate and evaluation.

Specifically,after obtaining the combined gradients from two views, this paper introduces an exploratory process of candidate and evaluation instead of simply choosing the edge with the most significant gradient as the perturbed edge for this iteration. Firstly, determine the size *S* of the candidate set. After sorting the momentum gradients, based on perturbation rules defined in Eq (6), *S* edges are sequentially selected to join the candidate set according to the edges’ actual existence and gradient saliency, forming a set of candidate perturbation edges. The candidate set H is represented as:

$$\begin{array}{c} H^{\left(k+1\right)}=\left(E_{1}^{\left(k+1\right)},E_{2}^{\left(k+1\right)},\cdots ,E_{S}^{\left(k+1\right)}\right). \end{array}$$ (13)

Secondly, each edge in the candidate set is individually added to the perturbation vector generated in the previous iteration, forming candidates for this iteration’s perturbation graph. All candidate perturbation graphs are then input into the evaluation function sequentially. The evaluation function used is as follows:

$$\begin{array}{c} Z=f_{{\left(\theta^{\prime }\right)}^{k}}\left(G_{i}^{\left(k+1\right)}\right), \end{array}$$ (14)

$$\begin{array}{c} Ev\left(G_{i}^{\left(k+1\right)}\right)=\sum_{v\in V_{U}}L\left(Z_{1},Z_{2}\right). \end{array}$$

*Z* is the embedding representation obtained through forward propagation of the model trained in the previous iteration, L is the contrastive loss value, and *Z*_1_ and *Z*_2_ represent the embedding representations of two views after passing through the shared graph network. The contrastive loss is computed, and the candidate edge with the worst loss is selected as the officially perturbed edge for this iteration. This selected edge is added to the previously generated perturbation vector, forming the perturbation graph for this iteration. The optimal perturbation graph is selected as follows:

$$\begin{array}{c} G_{a}^{\left(k+1\right)}=\underset{G^{\left(k+1\right)}}{\text{arg}\;\text{max}}\left(Ev\left(G_{1}^{\left(k+1\right)}\right),Ev\left(G_{2}^{\left(k+1\right)}\right),\cdots ,Ev\left(G_{S}^{\left(k+1\right)}\right)\right). \end{array}$$ (15)

$G_{a}^{\left(k+1\right)}$ represents the perturbed edge for this iteration, and $Ev(G_{i}^{\left(k+1\right)})$ is the evaluation result for each perturbation candidate graph. The candidate evaluation process is akin to comparing the importance of candidate perturbed edges in the graph structure. The overall algorithm process is shown in Algorithm 1

**Algorithm 1** MCGCL

**Input**: Original graph *G* = (*A*, *X*), differentiable encoder *f*, stochastic augmentation set *T*, number of perturbations *σ*, number of iterations *K*, momentum coefficient *p*,size of candidate set *S*.

**Output**: Poisoned graph *G* = (*A*′, *X*).

1: *i* = 0,*A*′ = *A*.

2: **while** *i* < *σ* **do**

3:  Train *f* with *A*′ and *X*.

4:  Δ′ = 0.

5:  **for** *k* = 1 to *K* **do**

6:   Sample two stochastic augmentations $t_{1}^{k},t_{2}^{k}\in T$.

7:   Obtain two views($A_{1}^{k},X_{1}^{k})=t_{1}^{k}$ (*A*′, *X*), $(A_{2}^{k},X_{2}^{k})=t_{2}^{k}$ (*A*′, *X*).

8:   Forward propagate($A_{1}^{k},X_{1}^{k}$),($A_{2}^{k},X_{2}^{k}$) through f and compute contrastive loss L.

9:   Obtain the gradients of $A_{1}^{k}$ and $A_{2}^{k}$ w.r.t. the contrastive loss, $\Delta_{1}^{k}=\frac{\partial L}{\partial A_{1}^{k}}$, $\Delta_{2}^{k}=\frac{\partial L}{\partial A_{2}^{k}}$.

10:   Compute momentum gradients of $A_{1}^{k}$ and $A_{2}^{k}$, $\Delta_{m1}^{k}=\Delta_{1}^{k}+p\Delta_{m1}^{\left(k-1\right)}$, $\Delta_{m2}^{k}=\Delta_{2}^{k}+p\Delta_{m2}^{\left(k-1\right)}$.

11:   Sum the gradients of two views, $\Delta^{\prime }=\Delta^{\prime }+\Delta_{m1}^{k}$ + $\Delta_{m2}^{k}$.

12:  **end for**

13:  Sort in descending order of absolute gradient value.

14:  **for** *s* = 1 to *S* **do**

15:   Compute the [row, column] values of the edge corresponding to the gradient values in the adjacency matrix.

16:   **if** *A*′[*a*, *b*] = 1, Δ′[*a*, *b*] < 0 or *A*′[*a*, *b*] = 0, Δ′[*a*, *b*] > 0 **then**

17:    Form the perturbed candidate graph of the current iteration by adding [a,b] to the perturbed graph formed in the last iteration.

18:    Calculate the loss of each candidate graph by evaluation function.

19:   **end if**

20:  **end for**

21:  Compare and select the edge [*m*, *n*] with the highest loss as the perturbation edge of this iteration.

22:  *A*′[*m*, *n*] = 1-*A*′[*m*, *n*].

23:  Freeze the chosen edge and avoid being flipped again in next iterations.

24:  *i* = *i* + 1.

25: **end while**

### Complexity analysis

#### Time complexity

Firstly, assuming the complexity of the forward propagation process in the GCA model is denoted as *O*(|*E*|). The time complexity for retraining the contrastive model once is denoted as *O*(*K*|*E*|), where *K* represents the number of iterations for training the model. The MCGCL method’s computational costs mainly involve generating momentum gradients, gradient sorting, and candidate evaluation. The process of generating momentum gradients can be considered recursive, and the complexity of weighted summation is constant, resulting in a time complexity denoted as *O*(*σ*). The time complexity for gradient sorting is *O*(|*V*|^2^), and for candidate evaluation, it is *O*(*S*|*E*|). Therefore, the total time complexity of MCGCL is denoted as *O*(*K* + *S*)*σ*|*E*| + *O*(*σ*) + *O*(*σ*|*V*|^2^).

Although the complexity of the gradient sorting process is denoted as *O*(|*V*|^2^), which mainly involves a straightforward sorting operation, the primary computational costs still lie in other parts. Disregarding the differences in surrogate model structure and complexity, leading to significant variations in computational efficiency due to retraining the model, the inherent computational efficiency of the MCGCL method is similar to that of the Metattack [16]. Compared with GLGA [29], the primary computational costs occur during the candidate evaluation stage. On the one hand, the additional costs have a negligible impact on improving the attack success rate. On the other hand, the trade-off between attack effectiveness and computational efficiency can be balanced by adjusting the size of the candidate set. For Min-Max [17], during poisoning attacks, it is necessary to retrain the model and utilize random sampling to generate each perturbation, resulting in additional computational overhead.

#### Space complexity

Compared to CLGA [29], due to the need to calculate the adjacency matrix gradient, the memory requirement is *O*(*N*^2^), where *N* represents the number of nodes in the graph. Additionally, each iteration requires saving lists of candidate perturbation graphs and evaluation function results, with both sizes equal to the size of the candidate set *S*. However, MCGCL saves candidate perturbed edges rather than the entire candidate perturbation graph to reduce memory consumption. After each iteration of candidate perturbation is completed, the memory is immediately cleared and released, thereby reducing the memory cost. The Metattack [16] incurs significant storage space consumption while generating meta-gradients.

Taking into account the time and space costs of the MCGCL method, they are both within manageable limits. Simultaneously, adjusting the candidate set size allows for a balance between computational efficiency and attack effectiveness compared to other baseline methods. Using momentum gradients for parameter updates accelerates convergence, reduces the number of iterations required for model training, and improves attack effectiveness.

## Experiments

### Setup

#### Datasets

This paper employs the Cora, CiteSeer, and PolBlogs datasets. Cora and CiteSeer datasets are citation networks where each node has corresponding attribute features, while the PolBlogs dataset is a social network graph with nodes lacking features. The basic details of the datasets are summarized in Table 2.

**Table 2:** The basic details of the datasets.

Dataset	Nodes	Edges	Features	Classes
**Cora**	2708	5278	1433	7
**CiteSeer**	3327	4552	3703	6
**PolBlogs**	1490	16715	None	2

#### Baselines

This paper focuses on global poisoning attacks. The supervised baseline methods employed include DICE [44], PGD [17], Min-Max [17], and Metattack [16]. CLGA [29] and Bojchevski [27] are the unsupervised baseline methods mentioned.

#### Experimental settings

The baseline experimental data in this paper is sourced from CLGA [29]. For Metattack [16], PGD [17], and Min-Max [17] baseline methods, a two-layer GCN model is employed as the surrogate model. All attack methods involve initially generating perturbation graphs, which are then fed into the advanced graph-contrastive learning model GCA [42] for training and model accuracy testing. The GCA model uses a two-layer GCN as the encoder. To enhance the representativeness and reliability of the experimental results while reducing random errors, the paper conducts ten experiments separately for node classification and link prediction tasks, taking the average as the final result.

In this paper, the perturbation rates were set to 1%/5%/10% of the total number of edges in the original graph. We use grid search to determine the optimal value of hyperparameters, i.e. the candidate set sizes are chosen from {64, 96, 128}, and momentum coefficients are selected from {0.75, 0.8, 0.85, 0.9}. The optimal sizes for the candidate set and momentum coefficient are 128 and 0.85, respectively. For ease of comparing the effects of different attack strategies, the paper sets the hyperparameters of the GCA [42] model in the experiments, including temperature coefficient *τ* and random augmentation rate for data, to fixed values. Expressly, the temperature coefficient *τ* is set to 0.4, the topological augmentation rates for two views are set to 0.3 and 0.4, the feature augmentation rate is set to 0.1 and 0.0, and the optimizer used is Adam. The learning rate is set to 0.01. For the PolBlogs dataset, 32-dimensional vectors are experimentally generated randomly and used as node features as nodes lack feature vectors.

For the node classification task, the Cora and CiteSeer datasets partitioning follows the publicly available split provided by Yang et al. [45]. For the PolBlogs dataset, we divided the nodes into training, validation, and test sets with a ratio of 10%/10%/80%, respectively. The learned embedding representations are utilized as inputs to a logistic regression model, and the classification accuracy is calculated.

For the link prediction task, the three datasets partition the edges into train/test/val sets with a ratio of 70%/20%/10%. A 2-layer MLP is employed as the projection head to map the learned embeddings into a new latent space. The MLP is trained using negative sampling and margin loss for edge prediction, and uses AUC values to test the performance of model link prediction.

### Experimental results and analysis of node classification

Table 3 presents the experimental results for node classification. Observing the table, except for the case on the Cora dataset, where the results are somewhat moderate when the perturbation rate is 0.01, our proposed method exhibits outstanding performance under other perturbation attacks. It significantly reduces the model’s accuracy in node classification tasks, generally surpassing other unsupervised methods.

**Table 3:** Results of joint test experiments for node classification.

	Attack	Cora			CiteSeer			PolBlogs		
		1%	5%	10%	1%	5%	10%	1%	5%	10%
Supervised	Metattack [16]	0.7586	**0.6928**	**0.6168**	**0.5920**	**0.3986**	**0.2952**	0.8208	0.8039	0.8011
	PGD [17]	0.7680	0.7592	0.7402	0.6098	0.6198	0.6056	0.8100	0.8010	0.7987
	Min-Max [17]	0.7624	0.7218	0.6174	0.6302	0.5254	0.5618	**0.8016**	0.7913	0.7986
	DICE [44]	0.7712	0.7642	0.7240	0.6256	0.5774	0.5246	0.8107	**0.7847**	**0.7394**
Unsupervised	Bojchevski [27]	0.7490	0.7710	0.7670	0.6442	0.6448	0.6608	0.8187	0.8042	0.7892
	CLGA [29]	**0.7316**	0.7188	0.6814	0.6368	0.5906	0.5368	0.8088	0.7944	0.7726
	MCGCL(ours)	0.7446	0.7002	0.6504	0.6176	0.5762	0.5206	0.8023	0.7864	0.7573

The boldfaced ones are the best in all methods, and the underlined ones are the best in unsupervised methods.

### Experimental results and analysis of link prediction

Table 4 presents the experimental results for link prediction. Observing the experimental data in the link prediction task, the Cora dataset exhibits performance comparable to or even better than supervised learning results when the perturbation rates are 0.05 and 0.1. Similarly, in the case of the CiteSeer dataset with a perturbation rate of 0.05, the method performs exceptionally well. For the PolBlogs dataset, except for the perturbation rate 0.01, our proposed method outperforms other unsupervised adversarial attack methods in link prediction. The experimental results demonstrate the effectiveness of our proposed attack method in link prediction tasks.

**Table 4:** Results of joint test experiments for link prediction.

	Attack	Cora			CiteSeer			PolBlogs		
		1%	5%	10%	1%	5%	10%	1%	5%	10%
Supervised	Metattack [16]	**0.9010**	0.8733	0.8500	**0.9109**	0.8853	**0.8544**	0.8617	0.8585	0.8635
	PGD [17]	0.9143	0.9073	0.9073	0.9169	0.9248	0.9057	0.8605	0.8584	0.8625
	Min-Max [17]	0.9116	0.9004	0.8944	0.9145	0.8890	0.8981	0.9145	0.8890	0.8981
	DICE [44]	0.9046	0.8828	0.8593	0.9137	0.8918	0.8679	**0.8551**	**0.8450**	**0.8352**
Unsupervised	Bojchevski [27]	0.9164	0.9099	0.9101	0.9239	0.9168	0.9196	0.8593	0.8543	0.8587
	CLGA [29]	0.9012	0.8741	0.8420	0.9114	0.8911	0.8610	0.8584	0.8598	0.8563
	MCGCL(ours)	0.9050	**0.8684**	**0.8356**	0.9124	**0.8828**	0.8583	0.8589	0.8493	0.8432

The boldfaced ones are the best in all methods, and the underlined ones are the best in unsupervised methods.

### Ablation experiment

This paper conducted ablation experiments to compare the impact of the two proposed strategies on the attack effectiveness. The momentum gradient (MCGCL_M) and candidate and evaluation (MCGCL_C) methods represent ablation experiments conducted using only the momentum gradient and the candidate evaluation method, respectively.

#### Momentum gradients experiment

The experimental results of MCGCL_M are presented in Table 5. Observing the experimental data, it can be noted that the generation of adversarial samples using the momentum gradient method alone, except for suboptimal performance in individual experiments, still demonstrates excellent overall attack effectiveness, highlighting the advantages of the momentum gradient.

**Table 5:** Experimental results of momentum gradients.

	Attack	Cora			CiteSeer			PolBlogs		
		1%	5%	10%	1%	5%	10%	1%	5%	10%
Node classification	MCGCL_M	0.7449	0.7075	0.6512	0.6273	0.5762	0.5232	0.7998	0.7912	0.7632
Link prediction	MCGCL_M	0.8964	0.8723	0.8379	0.9109	0.8872	0.8594	0.8590	0.8523	0.8473

#### Candidate and evaluation experiment

The experimental results of MCGCL_C are presented in Table 6. Observing the experimental data, it can be noted that the overall perturbation effect is good, especially in link prediction tasks with perturbation rates of 0.05 and 0.1 on the Cora dataset and with a perturbation rate of 0.05 on the CiteSeer dataset. MCGCL_C demonstrates better performance than supervised learning in these scenarios. The candidate evaluation method evaluates multiple candidate graphs in the candidate set, avoiding information errors introduced by the most significant gradient values and reducing the risk of the attack results falling into local optima.

**Table 6:** Experimental results of candidate and evaluation.

	Attack	Cora			CiteSeer			PolBlogs		
		1%	5%	10%	1%	5%	10%	1%	5%	10%
Node classification	MCGCL_C	0.7425	0.7038	0.6672	0.6294	0.5882	0.5243	0.8017	0.7885	0.7653
Link prediction	MCGCL_C	0.9052	0.8693	0.8282	0.9212	0.8838	0.8575	0.8596	0.8517	0.8459

From the overall experimental results, on the one hand, the parameter settings (including momentum coefficient and candidate set size) leading to good performance are dataset and downstream task-specific. At the same time, when the downstream task is link prediction, our proposed method shows results comparable to or even better than supervised learning, especially on the Cora dataset. On the other hand, the results from ablation experiments demonstrate that certain individual methods may outperform joint testing results in specific scenarios (such as MCGCL_C in link prediction tasks on the Cora and CiteSeer datasets at a perturbation rate of 0.1). Overall, joint testing results still exhibit superior performance. The experiments also validate that changes affecting model prediction are more inclined towards adding edges rather than removing edges. This is because adding edges can alter the original graph’s topological structure while removing edges results in the loss of information from the original graph, a conclusion supported by the findings in literature [19].

### Transferability analysis

On the Cora dataset, the MCGCL method uses the GCA [42] model to generate adversarial samples, which are then used to attack graph neural networks with different structures, such as GCN [39] and DeepWalk [28] models. A comparison is made with the overall best-performing supervised attack method, Metattack, and two other unsupervised attack methods to validate the transferability of the proposed method. Experimental results are presented in Tables 7 and 8.

**Table 7:** Experimental results of node classification task on classical graph geural networks.

Attack	Node Classification					
	DeepWalk			GCN		
	1%	5%	10%	1%	5%	10%
Metattack [16]	0.6766	**0.6370**	**0.5554**	**0.7830**	**0.7300**	**0.6330**
Bojchevski [27]	0.6826	0.6436	0.6276	0.8240	0.7900	0.7950
CLGA [29]	0.6720	0.6459	0.6376	0.7860	0.7770	0.7690
MCGCL(ours)	**0.6683**	0.6441	0.6293	0.7847	0.7538	0.7432

The boldfaced ones are the best in all methods, and the underlined ones are the best in unsupervised methods.

**Table 8:** Experimental results of link prediction task on classical graph neural networks.

Attack	Link prediction					
	DeepWalk			GCN		
	1%	5%	10%	1%	5%	10%
Metattack [16]	**0.8777**	0.8629	**0.8319**	**0.8655**	**0.8378**	0.8182
Bojchevski [27]	0.8852	0.8873	0.8810	0.8675	0.8634	0.8542
CLGA [29]	0.8799	0.8612	0.8390	0.8667	0.8546	0.8146
MCGCL(ours)	0.8780	**0.8602**	0.8342	0.8670	0.8426	**0.8113**

The boldfaced ones are the best in all methods, and the underlined ones are the best in unsupervised methods.

For both node classification and link prediction tasks, evaluation is performed using classification accuracy and AUC. Other experimental settings remain consistent with the experiments above. Observing Tables 7 and 8, it is evident that even though the Bojchevski attack method is specifically designed for the DeepWalk model, the MCGCL method still achieves significant attack effectiveness against the DeepWalk model, surpassing Bojchevski, particularly in link prediction. In attacking the GCN model, the MCGCL method demonstrates superior attack effectiveness compared to other unsupervised methods. The experimental results substantiate that the proposed method, employing generated perturbation graphs to attack diverse graph neural network architectures, significantly reduces model prediction accuracy, thereby validating the method’s efficacy and robust transferability.

### Visualization analysis

The results in Tables 3 and 4 show that the Metattack method exhibits the best overall performance. Therefore, in this paper, we conduct a comparative analysis of the embedding scatter plots between the Metattack and the proposed methods.

**Fig 2:**
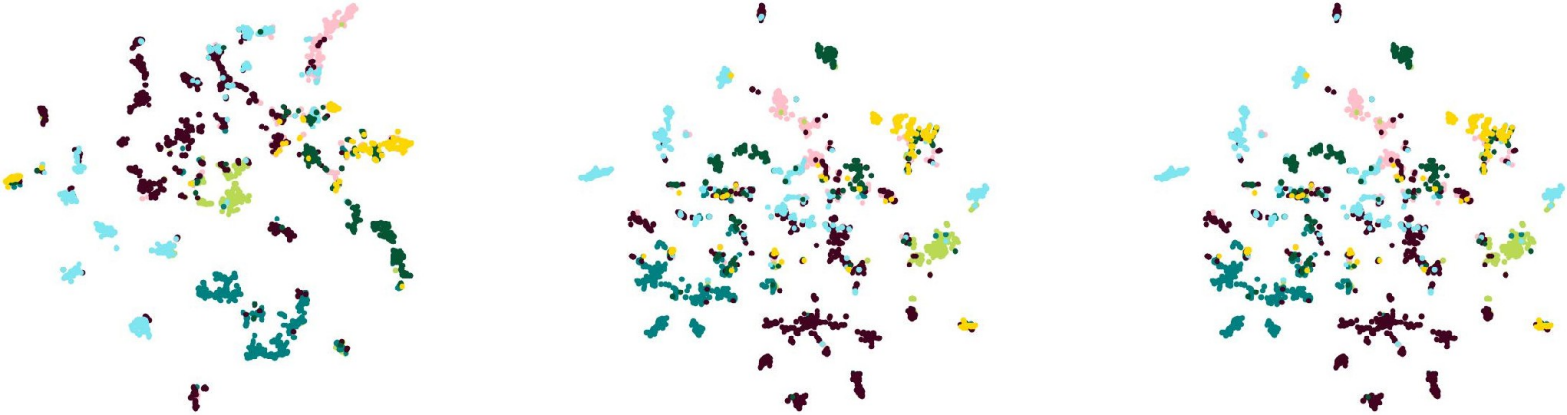
Embedding visualization scatter plots. From left to right are the embedded visualization scatter plots of clean graph, Metattack method, and MCGCL method, respectively.

**Fig 3:**
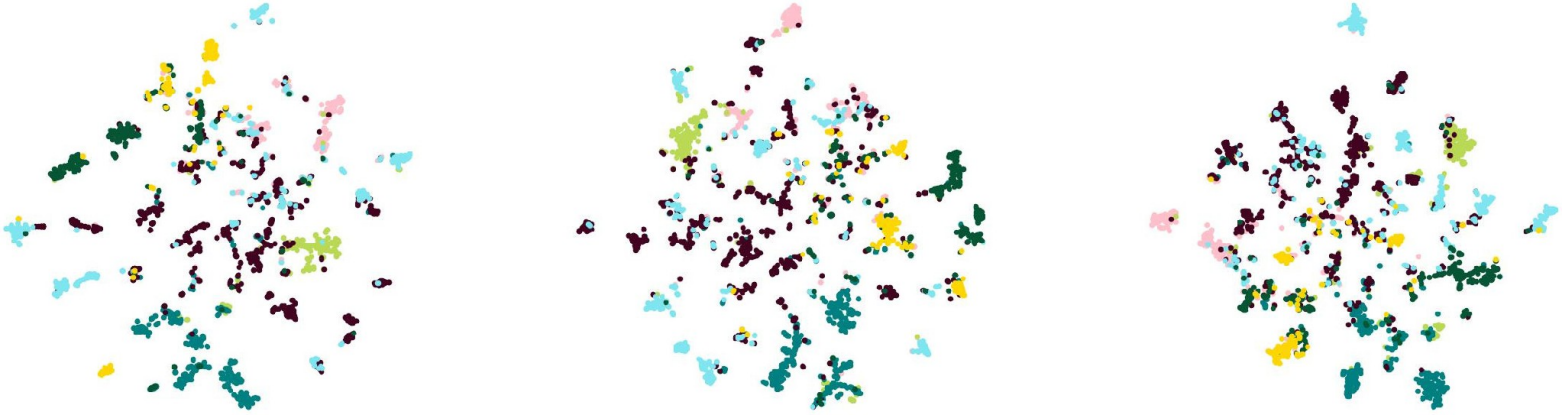
Embedding visualization scatter plots with different perturbation rates. From left to right are the embedded visualization scatter plots with perturbation rates of 0.01, 0.05, and 0.1, respectively.

Fig 2 illustrates the visual scatter plot of the embeddings of the attacked model after generating perturbation graphs through different perturbation methods. On the other hand, Fig 3 displays the visual scatter plot of the node embeddings generated by the MCGCL method under different perturbation rates after passing through the GCA model. It can be observed from the figures that perturbation graphs with introduced noise reduce the model’s predictive capability, causing the boundaries between different categories to become blurred and pulling nodes towards the center. Due to the similarity in distance between central nodes and each category results in a decrease in the model’s predictive generalization ability.

## Discussion

In the context of unsupervised attacks, the MCGCL method proposed in this paper uniquely addresses gradient reliability and saliency issues. By incorporating momentum gradients, the accuracy of gradient updates is enhanced. Simultaneously, leveraging candidate evaluation methods mitigates the problem of suboptimal attack outcomes resulting from reduced saliency of gradients. These effectively improve the attack accuracy.

### Discussion of experimental results

Due to the utilization of labels as additional knowledge in the other four supervised baseline attacks, they are expected to perform better than unsupervised attacks. However, experimental results from Tables 3 and 4 demonstrate that MCGCL shows comparable performance, and in some cases, it even outperforms specific supervised baselines.

Simultaneously, for the PGD [17], Min-Max [17], and Bojchevski [27] methods, the classification accuracy does not continuously decrease with the increasing perturbation rates. This suggests that these three methods could have effectively targeted the edges crucial for the model predictions, indicating the robustness of graph contrastive learning against these attacks. In contrast, other baseline methods improve attack effectiveness with increasing perturbation rates. However, Metattack [16] and DICE [44] methods heavily rely on labels, whereas this paper, building upon the CLGA [29], addresses issues arising from reduced gradient reliability and saliency. Consequently, our method achieves overall superior attack effectiveness.

### Limitations and mitigation methods

Due to the message passing mechanism of Graph Neural Networks (GNNs), the derivation process of structural gradients involves node features. This results in the propagation of noise from node features to structural gradients. Concurrently, graph contrastive learning effectively harnesses the advantages of topological and feature-level data augmentation, skillfully mitigating the structural gradient noise arising from node feature perturbations.

In addition to the saliency of gradients, factors influencing the attack results falling into local optima may also involve the greedy strategy in sequentially selecting perturbed edges while generating perturbation graphs, where the generation processes of perturbed edges may mutually interfere. Therefore, building upon candidate evaluation, this paper contemplates the potential effectiveness of mitigating the risk of attack results settling into local optima by introducing a more extensive candidate domain. An additional recombination phase is introduced to expand the candidate domain, aiming to reduce the interference in the generation process of perturbed edges. The next step involves rigorous experiments to validate this proposition.

## Conclusion

In this paper, within the context of unsupervised graph contrastive learning adversarial attacks based on gradients, we propose an exploratory method using momentum gradient candidates to address the local optima problem arising from the unreliability and saliency of gradients. The method’s effectiveness is validated across different datasets, downstream tasks, and perturbation rates. Experimental results demonstrate that the proposed method outperforms other unsupervised methods regarding attack effectiveness. It even surpasses supervised baseline methods in some link prediction experiments, exhibiting superior performance. This research accelerates convergence speed, enhances the accuracy of parameter updates, and consequently improves attack effectiveness. Additionally, the strong transferability of the proposed method is empirically demonstrated through experiments.

In future work, in terms of adversarial attacks, we plan to alleviate the problem of local optima caused by the sequential selection of perturbed edges proposed in the second section of the “Discussion” by expanding the candidate range to obtain a wider range of choices, and demonstrate the effectiveness of this method through experiments. More importantly, we plan to investigate defense strategies against attacks in graph contrastive learning. Currently, most defense strategies are primarily designed for supervised attacks, utilizing additional label information for model retraining and applying metrics such as degree centrality to prune malicious edges. Our future research direction will concentrate on proposing defense strategies tailored explicitly for countering unsupervised attacks. This aims to reduce dependence on labels, thereby enhancing the model’s robustness by acquiring minimal information.
